# Sennine in Eczema and Venerial Ulcers

**Published:** 1893-12

**Authors:** W. R. Hardesty


SENNINE IN ECZEMA AND VENERIAL ULCERS.—Eureka Springs, Ark., Oct, 9, 1893.—Dios Chemical Co., St. Louis, Mo.,—Gentlemen :—The sample of Sennine you sent me came safely to hand, and I happened to have some cases that visited my office daily for treatment. In two cases of eczema covering the inner side of thigh I applied the Sennine just as I received it from you, that is full strength, dry, and I am happy to say it acted like a charm in both cases. Again I applied Sennine to venereal ulcer and must say that it did all any one could ask. I look upon Sennine as the antiseptic of all others and shall continue its use in my practice.

W. R. HARDESTY, M. D.

